# Treatment outcomes of non‐curative endoscopic submucosal dissection for superficial gastric neoplasia: A retrospective study at a tertiary care center in the United States

**DOI:** 10.1002/deo2.70034

**Published:** 2024-11-08

**Authors:** Sayaka Nagao, Makoto Nishimura, Mako Koseki, Jacques Beauvais, Monika Laszkowska, Laura Tang, Vivian E. Strong, Mark A. Schattner

**Affiliations:** ^1^ Gastroenterology, Hepatology, and Nutrition Service, Department of Medicine Memorial Sloan Kettering Cancer Center New York USA; ^2^ Department of Pathology and Laboratory Medicine Memorial Sloan Kettering Cancer Center New York USA; ^3^ Department of Surgery Memorial Sloan Kettering Cancer Center New York USA

**Keywords:** dysplasia, early gastric cancer, endoscopic submucosal dissection, lymph node metastasis, treatment outcome

## Abstract

**Objectives:**

Endoscopic submucosal dissection (ESD) is a minimally invasive treatment for early gastric cancer; additional treatment may be recommended for patients in whom resection is not curative per the American Society for Gastrointestinal Endoscopy guidelines. The aim of this study was to assess treatment outcomes of ESD for gastric neoplasia, with a focus on cases of non‐curative resection.

**Methods:**

This was a retrospective study of all individuals undergoing ESD for the treatment of gastric adenocarcinoma or dysplasia in a high‐volume tertiary care center in the United States. Data on patient demographic characteristics, clinical history, lesion characteristics, and procedural and clinical outcomes were collected from the electronic medical record.

**Results:**

Among 82 cases undergoing ESD for the management of gastric neoplasia, 32 cases resulted in non‐curative resection. 20 of these non‐curative cases did not get additional treatment, among which recurrence occurred in two cases with positive horizontal margins only. These patients did not show lymph node metastasis and underwent further endoscopic or surgical resection. There was no recurrence in 11 cases with undifferentiated carcinomas of ≤2 cm in size.

**Conclusions:**

Although this study was limited by its retrospective design, small sample size, and follow‐up duration, our findings suggest that a risk‐adapted strategy could be employed for certain patients undergoing non‐curative ESD per American Society for Gastrointestinal Endoscopy guidelines, with close follow‐up instead of routine surgery in select cases with favorable features. Further studies are needed to refine the criteria for additional treatment after non‐curative ESD in Western populations.

## INTRODUCTION

Endoscopic submucosal dissection (ESD) is a minimally invasive treatment for early gastric cancer (EGC)^1^, ^2^ that has become standard practice in Eastern countries^3^ and is gaining popularity in the United States.^4^ Although ESD can be curative in cases without lymph node metastasis (LNM),^5^, ^6^, ^7^, ^8^ additional treatment is recommended in cases with histopathological features indicating a high risk of metastasis,^9^ that is, in cases in which ESD is non‐curative. However, the rate of LNM in patients who undergo surgery after non‐curative ESD is relatively low, suggesting that many patients may undergo unnecessary additional surgery.^10^ Thus, determining the appropriate criteria for additional surgery is an important but major challenge.

The American Society for Gastrointestinal Endoscopy (ASGE) recently published guidelines for ESD in cases of early esophageal and gastric cancers.^4^, ^11^ It defined curative resection as the absence of malignant cells at the lateral and deep margins (R0 resection), well or moderately differentiated histology, and no lymphovascular or deep invasion beyond the submucosa. Although surgical referral is generally recommended in non‐curative cases, the guidelines emphasize the importance of a multidisciplinary review that considers the risks associated with gastrectomy. The guidelines suggest an approach for weighing the advantages and disadvantages of further treatment versus observation in each case. In contrast, the Japanese Gastric Cancer Association (JGCA)^3^ and European Society of Gastrointestinal Endoscopy guidelines^12^ do not recommend additional treatment, even for undifferentiated carcinoma cases, if they meet specific criteria including no ulcerative findings, en bloc resection, pT1a lesion, negative horizontal and vertical margins, no lymphovascular infiltration, and tumor size of ≤2 cm, as the predicted risk of LNM is low in such cases. There have been few reports on gastric ESD outcomes in the US population.^13^, ^14^ Hence, to help inform the decision‐making process for additional treatment, in this study, we aimed to evaluate the treatment outcomes in non‐curative resection cases of gastric neoplasia at our institution, which is a high‐volume referral center for ESD in the United States.

## METHODS

### Definition of curative resection

All the resected lesions were histologically diagnosed by a specialized gastrointestinal pathologist. The location, macroscopic and histological types, and invasion depth were defined according to the American Joint Committee on Cancer TNM staging (AJCC Cancer Staging Manual 8th Edition^15^) or the Japanese Classification of Gastric Carcinoma by the JGCA.^16^ Macroscopic types of gastric neoplasia were further classified into three groups: elevated (Type 0–I or Type 0–IIa), flat (Type 0–IIb), and depressed (Type 0–IIc or Type 0–III).^17^ Invasion depth was classified into intramucosal cancer (M), cancer with submucosal invasion < 500 µm (SM1), and cancer with submucosal invasion ≥ 500 µm (SM2). Resection was considered en bloc if a lesion was retrieved from a single specimen. Resection was considered R0 whenever the pathological evaluation showed a negative horizontal and vertical margin (HM0 and VM0, respectively). Curative resection was defined according to the ASGE guidelines as the absence of malignant cells at the lateral and deep margins, well or moderate differentiation, and no lymphovascular or deep invasion beyond the submucosa.^4^ 500 µm is the limit for invasion depth considered curative for gastric adenocarcinoma. Cases that did not meet these criteria for curative resection were defined as non‐curative.

Residual disease was defined as the presence of neoplastic tissue, confirmed by pathological evaluation during endoscopic treatment or surgery (residual parietal lesion), or as the detection of LNM in the surgical specimen. Lesions containing undifferentiated components were classified as undifferentiated carcinoma.

### Follow‐up after ESD

Before the recently established ASGE guidelines,^4^ the recommendations for follow‐up after ESD at our institution were to observe cases that met the curative criteria based on the JGCA guidelines,^3^ including en bloc resection, HM0, and VM0, without lymphovascular infiltration, in addition to 1) differentiated type‐dominant, pT1a, without ulcerative findings, and any tumor size, 2) undifferentiated type‐dominant, pT1a, without ulcerative findings, and tumor size ≤2 cm, and 3) differentiated type‐dominant, pT1a, with ulcerative findings, and tumor size ≤3 cm, or 4) differentiated type‐dominant, pT1b (SM1), and tumor size ≤3 cm. Additional treatment was recommended for all other cases. In cases where HM1 was the only factor for noncurative resection, observation was also an option.

Given the differences between the JGCA guidelines and the recent ASGE guidelines, in this study, we aimed to compare clinical outcomes in cases that would have been defined as non‐curative by the ASGE guidelines, although they were initially managed according to the JGCA guideline definitions.

### Study design and patient selection

Institutional review board approval was obtained for this study (protocol no. 20–495A). Individuals with ESD procedures completed between June 2018 and January 2024 for resection of gastric neoplasia who had a confirmed diagnosis of adenocarcinoma or dysplasia on pathology were included. Patients with esophagogastric junction lesions were excluded. Medical records were retrospectively reviewed and data regarding endoscopy and pathology findings were extracted.

The primary endpoint of this study was the outcome of patients classified as having a non‐curative resection according to ASGE guidelines who were initially managed according to the JGCA guidelines, to evaluate risk factors for LNM and/or residual disease in patients who received additional treatment and risk factors for recurrence in patients selected for observation.

### ESD procedures

ESD procedures were performed under general anesthesia or monitored anesthesia care with dedicated anesthesia staff. During this period, ESDs were performed using an EVIS EXERA III imaging platform (Olympus America), with GIF‐H 190 or GIF‐HQ190 endoscopes (Olympus America). The borderline diagnosis was performed with white‐light and narrow‐band imaging before ESD. Furthermore, submucosal injection was performed using saline or other available viscous solutions. Notably, a 1.5‐mm DualKnife J (Olympus America) or a 1.5‐, 2.0‐, or 3.0‐mm ORISE ProKnife (Boston Scientific) was used for cutting.

### Statistical analysis

Data were analyzed using JMP version 17 (SAS Institute Inc.). Categorical variables were described as absolute (*n*) and relative frequencies (%). For variables with missing data, available lesion analysis was performed. Fisher's exact test was used to assess the association between two categorical variables. All p‐values were two‐sided, and *p* < 0.05 was considered statistically significant.

## RESULTS

Between June 2018 and January 2024, 82 lesions with a diagnosis of gastric adenocarcinoma or dysplasia were treated with ESD at our institution. According to the ASGE guidelines, 50 of the 82 resections were identified as curative and 32 were non‐curative. The detailed clinical characteristics are shown in Table 1. There were no significant differences between the curative and non‐curative groups in terms of age, sex, race, lesion location, macroscopic type, or tumor size. No gastric neoplasia‐related deaths occurred during the study period.

**TABLE 1:** Baseline clinicopathologic characteristics of the patients.

	Curative resection	Non‐curative resection	
	*n* = 50	*n* = 32	*p*‐value
Sex, *n* (%)			0.170
Female	18 (36.0)	17 (53.1)	
Male	32 (64.0)	15 (46.9)	
Age, *n* (%)			0.053
<65 years	12 (24.0)	15 (46.9)	
≥65 years	38 (76.0)	17 (53.1)	
Race, *n* (%)			0.204
White	25 (50.0)	22 (68.8)	
Asian	21 (42.0)	10 (31.3)	
Black or African American	3 (6.0)	0 (0)	
Other Race	1 (2.0)	0 (0)	
Location, *n* (%)			0.511
Upper	4 (8.0)	5 (15.6)	
Middle	23 (46.0)	17 (53.1)	
Lower	21 (42.0)	9 (28.1)	
Remnant	2 (4.0)	1 (3.1)	
Macroscopic type, *n* (%)			0.075
Elevated	22 (44.0)	9 (28.1)	
Flat	0 (0)	2 (6.3)	
Depressed	28 (56.0)	21 (65.6)	
Tumor size, *n* (%)			1.000
≤30 mm	45 (90.0)	30 (93.8)	
>30 mm	2 (4.0)	2 (6.3)	
Missing data, *n* (%)	3 (6.0)		
Histological type, *n* (%)			<0.001
Dysplasia	21 (42.0)	2 (6.3)	
Adenocarcinoma	29 (58.0)	30 (93.8)	
Differentiation, *n* (%)			<0.001
Differentiated	29 (100.0)	9 (30.0)	
Undifferentiated	0 (0)	21 (70.0)	
En bloc resection, *n* (%)			0.056
Yes	50 (100.0)	29 (90.6)	
Piecemeal	0 (0)	3 (9.4)	
Resection, *n* (%)			<0.001
R0	50 (100.0)	21 (65.6)	
R1	0 (0)	10 (31.3)	
Rx	0 (0)	1 (3.1)	
Horizontal margins, *n* (%)			<0.001
HM0	50 (100.0)	23 (71.9)	
HM1	0 (0)	8 (25.0)	
HMx	0 (0)	1 (3.1)	
Vertical margins, *n* (%)			0.007
VM0	50 (100.0)	27 (84.4)	
VM1	0 (0)	4 (12.5)	
VMx	0 (0)	1 (3.1)	
Invasion depth, *n* (%)			<0.001
M or dysplasia	46 (92.0)	17 (53.1)	
SM1	4 (8.0)	3 (9.4)	
>SM1	0 (0)	12 (37.5)	
Lymphovascular invasion, *n* (%)			<0.001
Positive	0 (0)	8 (25.0)	
Negative	50 (100.0)	24 (75.0)	

Abbreviations: M, mucosa; SM, submucosa.

Elevated, Type 0–I or Type 0–IIa; flat, Type 0–IIb; depressed, Type 0–IIc or Type 0–III.

Among the 32 non‐curative cases, 17 were found to have SM2 invasion, positive lymphovascular invasion (LVI), positive vertical margin, undifferentiated carcinoma larger than 2 cm, or SM1 and larger than 3 cm, and were recommended for additional treatment (Table 2). No significant differences were observed in clinical characteristics between the additional treatment and observation‐recommended groups, except for in LVI and invasion depth. Among the patients who required additional treatment, one received argon plasma coagulation, another received systemic chemotherapy at an outside hospital (regimen unknown), and 10 received additional surgical treatment. Five patients opted not to receive additional treatment and were carefully followed for recurrence.

**TABLE 2:** Comparison between patients who were recommended additional treatment and observation among non‐curative cases.

	Additional treatment recommended	Observation recommended	
	*n* = 17	*n* = 15	*p*‐value
Sex, *n* (%)			0.502
Female	8 (47.1)	9 (60.0)	
Male	9 (52.9)	6 (40.0)	
Age, *n* (%)			1.000
<65 years	8 (47.1)	7 (46.7)	
≥65 years	9 (52.9)	8 (53.3)	
Race, *n* (%)			1.000
White	12 (70.6)	10 (66.7)	
Asian	5 (29.4)	5 (33.3)	
Black or African American	0 (0)	0 (0)	
Other race	0 (0)	0 (0)	
Location, *n* (%)			1.000
Upper	2 (11.8)	3 (20.0)	
Middle	9 (52.9)	8 (53.3)	
Lower	5 (29.4)	4 (26.7)	
Remnant	1 (5.9)	0 (0)	
Macroscopic type, *n* (%)			0.102
Elevated	7 (41.2)	2 (13.3)	
Flat	0 (0)	2 (13.3)	
Depressed	10 (58.8)	11 (73.3)	
Tumor size, *n* (%)			0.486
≤30 mm	15 (88.2)	15 (100.0)	
>30 mm	2 (11.8)	0 (0)	
Histological type, *n* (%)			1.000
Dysplasia	1 (5.9)	1 (6.7)	
Adenocarcinoma	16 (94.1)	14 (93.3)	
Differentiation, *n* (%)			0.440
Differentiated	6 (37.5)	3 (21.4)	
Undifferentiated	10 (62.5)	11 (78.6)	
En bloc resection, *n* (%)			1.000
Yes	15 (88.2)	14 (93.3)	
Piecemeal	2 (11.8)	1 (6.7)	
Resection, *n* (%)			0.843
R0	10 (58.8)	11 (73.3)	
R1	6 (35.3)	4 (26.7)	
Rx	1 (5.9)	0 (0)	
Horizontal margins, *n* (%)			1.000
HM0	12 (70.6)	11 (73.3)	
HM1	4 (23.5)	4 (26.7)	
HMx	1 (5.9)	0 (0)	
Vertical margins, *n* (%)			0.069
VM0	12 (70.6)	15 (100.0)	
VM1	4 (23.5)	0 (0)	
VMx	1 (5.9)	0 (0)	
Invasion depth, *n* (%)			<0.001
M or dysplasia	3 (17.6)	14 (93.3)	
SM1	2 (11.8)	1 (6.7)	
>SM1	12 (70.6)	0 (0)	
Lymphovascular invasion, *n* (%)			0.003
Positive	8 (47.1)	0 (0)	
Negative	9 (52.9)	15 (100.0)	
Additional treatment, *n* (%)			
Surgery	10 (58.8)		
Observation	5 (29.4)		
Chemotherapy	1 (5.9)		
APC	1 (5.9)		

Abbreviations: APC, argon plasma coagulation; M, mucosa; SM, submucosa.

Elevated, Type 0–I or type 0–IIa; flat, Type 0–IIb; depressed, Type 0–IIc or Type 0–III.

Among the 10 patients who underwent surgery, two patients showed LNM, and two had residual parietal lesions (Table 3). There were no significant differences in the clinical characteristics between patients with LNM or residual parietal lesions and those without residual lesions.

**TABLE 3:** Risk factors for residual lesions (residual parietal lesions and/or lymph node metastasis) in the additional surgery group.

	Residual parietal lesion and/or LNM		
	Yes (*n* = 4)	No (*n* = 6)	*p*‐value
Sex, *n* (%)			0.500
Female	2 (50.0)	1 (16.7)	
Male	2 (50.0)	5 (83.3)	
Age, *n* (%)			1.000
<65 years	2 (50.0)	3 (50.0)	
≥65 years	2 (50.0)	3 (50.0)	
Race, *n* (%)			1.000
White	3 (75.0)	4 (66.7)	
Asian	1 (25.0)	2 (33.3)	
Black or African American	0 (0)	0 (0)	
Other race	0 (0)	0 (0)	
Location, *n* (%)			1.000
Upper	0 (0)	0 (0)	
Middle	3 (75.0)	5 (83.3)	
Lower	1 (25.0)	1 (16.7)	
Remnant	0 (0)	0 (0)	
Macroscopic type, *n* (%)			0.571
Elevated	1 (25.0)	3 (50.0)	
Flat	0 (0)	0 (0)	
Depressed	3 (75.0)	3 (50.0)	
Tumor size, *n* (%)			0.467
≤30 mm	4 (100.0)	4 (66.7)	
>30 mm	0 (0)	2 (33.3)	
Histological type, *n* (%)			1.000
Dysplasia	0 (0)	0 (0)	
Adenocarcinoma	4 (100.0)	6 (100.0)	
Differentiation, *n* (%)			0.076
Differentiated	0 (0)	4 (66.7)	
Undifferentiated	4 (100.0)	2 (33.3)	
En bloc resection, *n* (%)			1.000
Yes	4 (100.0)	5 (83.3)	
Piecemeal	0 (0)	1 (16.7)	
Resection, *n* (%)			1.000
R0	3 (75.0)	3 (50.0)	
R1	1 (25.0)	2 (33.3)	
Rx	0 (0)	1 (16.7)	
Horizontal margins, *n* (%)			0.467
HM0	4 (100.0)	3 (50.0)	
HM1	0 (0)	2 (33.3)	
HMx	0 (0)	1 (16.7)	
Vertical margins, *n* (%)			0.667
VM0	3 (75.0)	5 (83.3)	
VM1	1 (25.0)	0 (0)	
VMx	0 (0)	1 (16.7)	
Invasion depth, *n* (%)			0.467
M or dysplasia	0 (0)	0 (0)	
SM1	0 (0)	2 (33.3)	
>SM1	4 (100.0)	4 (66.7)	
Lymphovascular invasion, *n* (%)			0.524
Positive	3 (75.0)	2 (33.3)	
Negative	1 (25.0)	4 (66.7)	
Lymph node metastasis, *n* (%)	2 (50.0)		
Residual parietal lesion, *n* (%)	2 (50.0)		

Abbreviations: LNM, lymph node metastasis; M, mucosa; SM, submucosa.

Elevated, Type 0–I or Type 0–IIa; flat, Type 0–IIb; depressed, Type 0–IIc or Type 0–III.

Of the 32 cases that would be defined as non‐curative according to the ASGE guidelines, 20 patients did not receive additional treatment after ESD and were followed up carefully for recurrence using endoscopy or computed tomography. The median observation period for this group was 24.9 months. Two patients showed recurrence and underwent additional ESD and/or surgery; local recurrence without LNM was confirmed in these patients. These two cases both had a positive horizontal margin in the initial ESD, whereas only two of 18 cases without recurrence had a positive horizontal margin (*p* = 0.032; Table 4). Notably, 100% (11/11) of the cases with undifferentiated, ≤2 cm, pT1a lesions that were classified as non‐curative resections according to the ASGE guidelines but classified as curative resections according to the JGCA guidelines did not have recurrence.

**TABLE 4:** Risk factors for recurrence in non‐curative cases without additional treatments.

	Recurrence		
	Yes (*n* = 2)	No (*n* = 18)	*p*‐value
Sex, *n* (%)			0.111
Female	0 (0)	13 (72.2)	
Male	2 (100.0)	5 (27.8)	
Age, *n* (%)			1.000
<65 years	1 (50.0)	9 (50.0)	
≥65 years	1 (50.0)	9 (50.0)	
Race, *n* (%)			0.521
White	2 (100.0)	11 (61.1)	
Asian	0 (0)	7 (38.9)	
Black or African American	0 (0)	0 (0)	
Other Race	0 (0)	0 (0)	
Location, *n* (%)			0.668
Upper	0 (0)	4 (22.2)	
Middle	2 (100.0)	7 (38.9)	
Lower	0 (0)	7 (38.9)	
Macroscopic type, *n* (%)			1.000
Elevated	0 (0)	4 (22.2)	
Flat	0 (0)	2 (11.1)	
Depressed	2 (100.0)	12 (66.7)	
Tumor size, *n* (%)			1.000
≤30 mm	2 (100.0)	18 (100.0)	
>30 mm	0 (0)	0 (0)	
En bloc resection, *n* (%)			0.100
Yes	1 (50.0)	18 (100.0)	
Piecemeal	1 (50.0)	0 (0)	
Histological type, *n* (%)			1.000
High‐grade dysplasia	0 (0)	1 (5.6)	
Adenocarcinoma	2 (100.0)	17 (94.4)	
Differentiation, *n* (%)			0.059
Differentiated	2 (100.0)	3 (17.6)	
Undifferentiated	0 (0)	14 (82.4)	
Resection, *n* (%)			0.053
R0	0 (0)	15 (83.3)	
R1	2 (100.0)	3 (16.7)	
Horizontal margins, *n* (%)			0.032
HM0	0 (0)	16 (88.9)	
HM1	2 (100.0)	2 (11.1)	
Vertical margins, *n* (%)			1.000
VM0	2 (100.0)	17 (94.4)	
VM1	0 (0)	1 (5.6)	
Invasion depth, *n* (%)			1.000
M or dysplasia	2 (100.0)	14 (77.8)	
SM1	0 (0)	1 (5.6)	
>SM1	0 (0)	3 (16.7)	
Lymphovascular invasion, *n* (%)			1.000
Positive	0 (0)	3 (16.7)	
Negative	2 (100.0)	15 (83.3)	

Abbreviations: M, mucosa; SM, submucosa.

Elevated, Type 0–I or Type 0–IIa; flat, Type 0–IIb; depressed, Type 0–IIc or Type 0–III.

## DISCUSSION

In this study, we retrospectively evaluated the treatment outcomes in ESD resection cases classified as non‐curative according to the ASGE guidelines and explored the risk factors for LNM or residual parietal lesions in patients who underwent additional surgery and those who did not. Among the 32 cases with non‐curative resection, 20 were followed by endoscopy or computed tomography without additional treatment, with local recurrence observed in two cases with positive horizontal margins on the original resection. No LNM or distant metastasis were observed during the study period. Therefore, follow‐up and additional treatment in cases of recurrence may be acceptable in patients with a positive horizontal margin as the only non‐curative factor.

Undifferentiated carcinoma is defined as non‐curative in the ASGE guideline.^4^ while it is defined as curative in the JGCA guideline^3^ if there are no ulcerative findings, pT1a, negative margins, no lymphovascular infiltration, and the tumor size is ≤2 cm. Reports from Japan have shown that the LNM rate in surgically resected EGC specimens meeting these criteria is 0% (0/310).^18^ In this study, 11 cases with undifferentiated, ≤2 cm, pT1a lesions were followed up and no recurrence was observed during the study period. Recent Western studies show promising outcomes for undifferentiated EGC treated with ESD, with one multicenter study reporting a 5.6% recurrence rate and no lymph node or distant metastasis,^19^ while another study specifically evaluating cases meeting JGCA criteria in North America found no recurrence, aligning with Japanese findings.^13^ Notably, one report from the United States noted LNM in 1/17 cases of intramucosal undifferentiated carcinomas of size ≤2 cm without ulcerative findings.^20^ These results suggest that among cases judged as non‐curative resections per the ASGE criteria, there may be cases with an extremely low risk of LNM in which ESD may still be appropriate. The decision to proceed with additional surgical treatment or pursue an observation strategy for these cases needs to be carefully evaluated.

The eCura scoring system, which scores histopathological findings after non‐curative ESD according to the JGCA guidelines, is known to predict the risk of LNM.^10^ According to the eCura system, the rates of LNM in the low‐, intermediate‐, and high‐risk groups were 2.5%, 6.7%, and 22.7%, respectively. Furthermore, in our study, the two patients who exhibited LNM after additional surgery had characteristics that would classify them in the intermediate‐risk group based on the eCura LNM risk assessment criteria. These patients had SM2 invasion with lymphatic invasion or positive vertical margins. However, our study has limitations in fully applying the eCura system. We assessed lymphovascular invasion as a combined factor; however, the eCura system requires separate evaluation of lymphatic and venous invasion for accurate scoring. Therefore, due to this difference in evaluation methods, we could not calculate the eCura score for all cases. Thus, although the eCura system can be used to predict LNM risk, further investigation is needed to validate its applicability in our patient population.

It is important to note that there are some reports that the frequency of LNM in patients with EGC in Western countries is higher than in Eastern countries.^20^, ^21^, ^22^ This intriguing phenomenon could be attributed to several factors. First, some studies have suggested biological differences in gastric cancer between different racial/ethnic groups may play a role in outcomes. For instance, Choi et al. reported that survival outcomes in T1a gastric cancers varied significantly by race.^21^ This could reflect underlying genetic or biological factors that influence tumor behavior and metastatic potential, potentially contributing to the observed differences in LNM rates between Western and Eastern populations. The results of this study did not show differences in treatment outcomes between races and suggest that overall treatment outcomes in Western populations are comparable to those in Eastern populations; however, larger future studies are needed to fully investigate these questions. Second, tumor location might be a contributing factor. Kumar et al. proposed that the location of the tumor might play a role in the differing LNM rates.^22^ In their series, patients with positive lymph nodes at surgery predominantly had proximal tumors. Gastric cancers in Western populations tend to be more proximal compared to those in Eastern populations, which could contribute to the higher LNM rates observed in Western studies. Third, differences in pathological criteria and interpretation may be an important factor. Hanada et al. highlight that there may be differences in pathological criteria and interpretation between Western and Asian pathologists.^20^ Western histologic criteria for intramucosal carcinoma emphasize invasion into the lamina propria, whereas Asian criteria also account for cytologic or architectural atypia that can be predictive of invasion. This difference in diagnostic thresholds could lead to some cases being classified differently between regions, potentially affecting the reported rates of LNM.

These factors underscore the complexity of comparing EGC outcomes across different populations and highlight the need for careful consideration when applying guidelines developed in one region to another. Further research is needed to elucidate these differences, potentially through large‐scale, multicenter studies with standardized pathological assessment protocols across Eastern and Western centers, and detailed molecular and genetic profiling of EGCs from different populations. Such studies will clarify whether observation alone can also be an acceptable treatment strategy for selected patients with non‐curative resection according to ASGE criteria, as in Japan, while considering the potential factors contributing to regional differences in LNM rates.

Strengths of this study include the examination of outcomes with detailed clinical data for patients who underwent noncurative resection according to ASGE criteria, which distinguishes it from previous larger multicenter studies that typically use JGCA criteria. However, this study has some limitations. First, our sample size of 32 non‐curative cases is relatively small, which limits our analyses. While this single‐center sample provides valuable insights, it may not be a full representation of the broader population. Second, the median follow‐up period of 24.9 months in this study provides valuable intermediate‐term data and is sufficient for detecting most recurrence. However, this timeframe does not allow for the assessment of long‐term outcomes such as 5‐year survival rates, which are standard measures in oncological studies. Third, the retrospective, single‐center design of our study may have introduced selection bias and limited the generalizability of our findings. However, despite these limitations, our study provides important data on the application of ASGE criteria in determining curative resection. Further prospective, multicenter studies with long‐term follow‐up data will be beneficial to comprehensively evaluate long‐term outcomes, including 5‐year survival rates, for patients managed without additional treatment after non‐curative ESD as defined by ASGE guidelines.

In conclusion, our study provides valuable insights into the outcomes of non‐curative ESD for gastric neoplasia in a Western population. We found that 10% of patients who were followed up after non‐curative ESD showed recurrence. Notably, cases with positive horizontal margins required careful follow‐up, as they were more likely to show regrowth of residual disease. However, these patients received additional treatment and showed no evidence of LNM or distant metastasis. Our findings suggest that careful follow‐up may be a feasible option for selected patients with positive horizontal margins as the only non‐curative factor, with the understanding that additional local treatment may be necessary if residual disease is detected. Furthermore, undifferentiated carcinomas with pT1a lesions, negative margins, no lymphovascular infiltration, and tumor size of ≤2 cm also showed no recurrence after ESD. Therefore, our findings highlight the need for case‐specific decision‐making in managing non‐curative ESD and highlight potential areas where ASGE guidelines could be refined. However, future large‐scale prospective studies are critical to validate these findings and develop more individualized, evidence‐based guidelines for non‐curative ESD management. Notably, such research will help optimize treatment strategies and improve outcomes for patients with early gastric neoplasia.

## CONFLICT OF INTEREST STATEMENT

Sayaka Nagao is a member of a laboratory that has received research funding from AI Medical Service Inc. This affiliation had no role in the preparation of the manuscript. Makoto Nishimura is a consultant for Boston Scientific, Olympus America, Fujifilm, Noah Medical, and AI Medical Service Inc. Monika Laszkowska and Makoto Nishimura received research funding from AI Medical Service Inc. Vivian E. Strong is a consultant for Regeneron and has received speaking honoraria from Merck. Mark A. Schattner is a consultant for Boston Scientific. The other authors declare no conflict of interest.

## ETHICS STATEMENT

Institutional review board approval was obtained for this study (protocol approval no. 20–495A).

## PATIENT CONSENT STATEMENT

The requirement for written informed consent from the patients was waived due to the retrospective nature of the study.

## CLINICAL TRIAL REGISTRATION

N/A.
